# Urinary oxytocin secretion after pituitary surgery, early arginine vasopressin deficiency and syndrome of inappropriate antidiuresis

**DOI:** 10.1007/s12020-024-04131-5

**Published:** 2024-12-17

**Authors:** Paul E. Constanthin, Nathalie Isidor, Sophie De Seigneux, Shahan Momjian

**Affiliations:** 1 Department of Neurosurgery, Hôpitaux Universitaires de Genève (HUG), Geneva, Switzerland; 2 Faculty of Medicine, Université de Genève (UNIGE), Geneva, Switzerland; 3 NeuroCentre, University Hospitals of Geneva, Geneva, Switzerland; 4 Department of Nephrology, Hôpitaux Universitaires de Genève (HUG), Geneva, Switzerland

**Keywords:** Pituitary surgery, Oxytocin, Arginine vasopressin deficiency, Hyponatraemia, Natriuresis, Central and postoperative diabetes insipidus

## Abstract

**Purpose:**

Transient arginine vasopressin deficiency (AVP-D), previously called diabetes insipidus, is a well-known complication of transsphenoidal pituitary surgery (TPS) with no definite predictive biomarker to date making it difficult to anticipate. While oxytocin (OXT) was previously suggested as a possible biomarker to predict syndrome of inappropriate diuresis (SIAD)-related hyponatraemia after TPS, its secretion in patients presenting with AVP-D remains poorly understood. We therefore hypothesized that OXT might present a different secretion in the case of AVP-D which would support its potential as an early biomarker of AVP-D. Moreover, we hypothesized that abnormal secretion of OXT might occur later on, notably with SIAD.

**Methods:**

We measured the urinary output of OXT in 67 consecutive patients subjected to TPS and compared the values of oxytocin between time-points and OXT ratio between groups. The primary endpoint of our study was to identify a difference in urinary OXT excretion in patients suffering from AVP-D compared to patients remaining normonatraemic. As a secondary endpoint, we compared the evolution of OXT secretion after the diagnosis of AVP-D in both groups, comparing the patients that later developed SIAD with the ones that did not.

**Results:**

Patients developing AVP-D showed a delay in the increase of OXT secretion after TPS as shown by a significantly lower ratio of OXT between the first postoperative day and the day of surgery (0.88 VS 1.68, p = 0.0162, IC:0.2979–0.2642) but a significantly higher ratio of OXT between the fourth and the first postoperative days (1.17 VS 0.53, p = 0.0006, IC:−2.109–0.6092). Moreover, normonatraemic patients that did not show normalization of OXT levels at day 4 after surgery tended to develop SIAD later on.

**Conclusion:**

Taken together, these results show for the first time that OXT release might help predict AVP-D after TPS and differentiate it from other pathologies of water-sodium balance.

## Introduction

Transsphenoidal pituitary surgery (TPS) is an established treatment for pituitary gland lesions [1]. Functional deficiency of the pituitary gland, also known as hypopituitarism, is a well-known complication of this surgery and might manifest early in abnormalities of the water-sodium balance, possibly leading to dysnatraemia [2–4]. One of those conditions in which water-sodium imbalance occurs after TPS is transient arginine vasopressin deficiency (AVP-D), previously known as diabetes insipidus [5], which might occur in up to 54% of the cases [3, 6, 7]. This condition is believed to be due to an abnormally low, or absent, pituitary release of arginine vasopressin (AVP) hormone [2, 8], leading to an increased loss of water that might ultimately result in hypernatraemia and hyperosmolality. Despite recent findings suggesting that copeptin, a byproduct of AVP synthesis, might help predict AVP-D by reflecting the postoperative secretion of AVP [9–15], copeptine measurement has not yet been demonstrated as sharply discriminative in the diagnosis of AVP-D after TPS, which remains based on clinical (daily quantification of both water intake and output to identify polydipsia/polyuria) and laboratory values (daily, or twice-daily, blood and urine analyzes to identify hypernatraemia and decreased Na concentration/osmolality of the urine) [3, 7, 16–18]. Its correct management (mainly through administration of the AVP-analog desmopressin (DDAVP) or fluid administration) can therefore prove challenging, notably in case of rapid onset of AVP-D [3, 16, 19]. Moreover, differentiating AVP-D from other conditions such as physiological or osmotic diuresis, of which clinical management is different from AVP-D, also remains difficult after TPS, further emphasizing the importance of the development of new biological markers [7, 16–18]. Lastly, correct identification of postoperative AVP-D is also particularly important due to the risk for these patients to develop permanent AVP-D (lasting longer than 6 months) later on [20]. Indeed, evidences have pointed to the existence of a triphasic course (AVP-D – syndrome of inappropriate antidiuresis (SIAD) – AVP-D) in postoperative dysnatraemia due to the reactions of the pituitary gland to the trauma of the surgery with, firstly, a sideration of the gland (responsible for early AVP-D), followed by an uncontrolled release of AVP (leading to SIAD-related hyponatraemia) and, finally, the loss of function of the neurohypophysis, resulting in permanent AVP-D [20, 21]. Therefore, early identification and treatment of those patients, to avoid any potentially chronic dysnatraemia, is of utmost importance.

AVP and its closely related hormone, oxytocin (OXT), are both produced in the paraventricular and supraoptic nuclei of the hypothalamus. They are then secreted through nervous terminals running to the posterior pituitary gland. In the last decades, OXT has been suggested to play a certain role in sodium balance [22–26]. Moreover, abnormal secretion OXT was observed in patients presenting central AVP-D, supporting the hypothesis that damage to AVP-producing cells might also result in lower OXT secretion [27–29]. Finally, in recent works, we observed an increased urinary secretion of OXT in patients later developing SIAD-related hyponatraemia after TPS [30] and correlated this increase in secretion with patients’ natriuresis [31]. [32–34] Therefore, we questioned whether OXT secretion, just like AVP, might be abnormal in patients developing AVP-D after TPS.

In this study, we analyzed the evolution of the urinary output of OXT in patients subjected to TPS, particularly comparing urinary OXT output between patients that developed AVP-D and the ones that remained normonatraemic. We hypothesized that OXT might present a different secretion in the case of AVP-D, which would support its potential as an early biomarker of AVP-D. Moreover, we hypothesized that abnormal secretion of OXT might occur later on, notably in the case of SIAD, as was reported in a previous study [30].

## Materials and methods

### Study design

This monocentric, observational study was conducted in a prospective way. Consecutive patients that were elected for transsphenoidal surgery for pituitary micro- and macro-adenomas or Rathke’s cleft cysts, between November 2015 and October 2022, were enrolled in the study at our center. Other inclusion criteria were for patients to be 18 y.o. or older and to be capable to give their free, informed consent to the research. The exclusion criteria were for the patients to be unable to give a free, informed consent. Basic patients’ information (age, biological sex…) as-well-as clinical information regarding tumor (size, presence or not of hormonal secretion) and pituitary function (presence or not of hypopituitarism requiring hormonal substitution) were obtained before surgery. Patients’ levels of sodium and osmolality in both blood and urine as-well-as their fluids intakes and output were followed daily during hospitalization and the presence of AVP-D was diagnosed clinically by the concomitant appearance of the following criteria: polyuria (>300 mL/hr for 2 h), plasma hyperosmolality (>300 mOsm/L), plasma osmolality higher than urine and hypernatraemia (≥145 mmol/l). Patients that presented with those criteria were diagnosed with post-TPS AVP-D and immediately treated orally with 1 dose of 0.1 mg or intravenously with 1 dose of 1 µg of desmopressin (DDAVP). If AVP-D persisted after this dose (persistence of hypernatraemia, blood hyperosmolality, urine hypo-osmolality and polyuria), a second dose of DDAVP was administered to the patients. No further confirmatory test (such as the hypertonic saline or AVP infusion tests) was performed before treatment. In the setting of our routine follow-up, Na urinary excretion was measured at the same time as OXT urinary excretion. All patients without further dysnatraemia were discharged on D7 post-surgery (which is the standard clinical practice after pituitary surgery in our department), while patients having been diagnosed with SIAD (criteria: increased urinary sodium concentration with urinary sodium concentration >30 mmol/L, hyponatraemia (<136 mmol/l), urine osmolality >100 mOsm/L and plasma osmolality lower than urine) were treated accordingly, with hydric restriction, until correction of hyponatraemia. Of note, as hyponatreamia and increased natriuresis appeared on D7, any clinical measures to correct natraemia (i.e. fluid restriction) had to be taken from that point in time, explaining the need for the patients to remain for a longer duration of hospitalization. Permanent AVP-D was diagnosed when treatment by DDAVP for AVP-D was required for longer than 6 months. If patients had a postoperative basal cortisol level on D4 below 400 nmol/l, they were maintained on 5 mg of prednisone per day to cover the time-period until a follow-up cortisolemia evaluation in the following weeks.

### Biological samples

Biological samples were analyzed as previously described [32]. Urine samples were obtained after an overnight fasting period on the day of the surgery and on days 1, 4, and 7 after the surgery. Samples were collected in prechilled plastic ethylenediaminetetraacetic acid tubes containing a proteinase inhibitor (Trasylol; Bayer, Leverkusen, Germany) and then centrifuged at 1300 *g* for 10 min at 4 °C. Samples were stored at −80 °C until analysis. Samples (0.5 ml) were kept at −20 °C until extraction using LiChroprep Si60 (Merck, Darmstadt, Germany) heat-activated at 700 °C for 3 h. Twenty milligrams of LiChroprep Si60 in 1 ml of distilled water was added to the sample, mixed for 30 min, washed twice with distilled water and 0.01 N HCl and eluded with 60% acetone. Extraction recovery was in the range 85–90%; data were not corrected for recovery. The lyophilized extract was then submitted to assay OXT using highly sensitive and specific radioimmunoassays (RIAgnosis, Munich, Germany). Although mainly used on plasma so far, the assay was strictly standardized and validated in many animal and human studies using a wide variety of stimuli (hypertonicity, parturition, lactation, stress, etc.) to reliably detect the bioavailable neuropeptides. Anti-OXT were raised in rabbits; as tracers, 125I-labeled neuropeptides (Perkin Elmer, Boston, MA, USA) were used. Assay sensitivities are in the 0.5-pg range, depending on the age of the tracers; cross-reactivities with related peptides, including AVP and DDAVP, are <0.7% and intra- and inter-assay variabilities are <10%. Using this assay, the basal urinary OXT levels (standardized to urinary creatinine as recommended in the literature) obtained in our patients are comparable to the basal values found in other studies using other assays [33, 34].

### Ethical approval

An informed consent was obtained from all patients included in the study. The ethical approval regarding blood and urinary samples (and their analyzes) and patients‘ data management was given by the local ethics committee of the Hôpitaux Universitaires de Genève (ethics committee number: 14–153). The entire study was conducted according to the approved protocol, which respected the Declaration of Helsinki regarding the ethical principles for medical research involving human subjects as-well-as the international recommendation for good clinical practices in clinical studies.

### Statistical analyzes

GraphPad Prism version 9 was used for statistical analysis. Descriptive statistics were expressed as median and quartiles (Q1; Q3). Fisher’s exact test was used when comparing qualitative values and Student’s unpaired t-test was used when comparing quantitative values. For the analyzes of urinary OXT, OXT values (standardized to concomitant creatinine value in the morning) on each day (day 0, day 1, day 4, and day 7) were compared between groups using an unpaired t-test. The evolution of OXT values was then compared, firstly for all patients (by pooling all results together), and then for each group (normonatraemic, AVP-D or SIAD patients), by repeated ANOVA with a Bonferroni-Holm correction for multiple testing, and by using a two-way ANOVA with a Bonferroni-Holm correction for multiple testing when comparing between groups. The ratios of OXT levels between successive days (D1/D0 and D4/D1) were compared between normonatraemic and AVP-D patients using Student’s unpaired t-test. A p-value lower than 0.05 was considered statistically significant.

## Results

### Groups

67 patients, 37 women (55.2%) and 30 men (44.8%), with a median age of 58 (48; 64.5) years old, were consecutively recruited based on magnetic resonance imaging (MRI). Regarding lesion type: 55 patients presented with a macroadenoma, 8 presented with a microadenoma, and 4 presented with a Rathke cleft cyst. Nineteen adenomas showed hormonal secretion: 11 secreted growth hormone (GH), 5 secreted adrenocorticotropic hormone (ACTH), 1 secreted both GH and prolactin (PRL), 1 secreted thyroid stimulating hormone (TSH) and 1 secreted follicle stimulating hormone (FSH). During the hospitalization, 13 patients were diagnosed with transient AVP-D in the first day after surgery and were treated with 1 (12 patients) or 2 (1 patient) doses of DDAVP. Twenty patients were later diagnosed with SIAD on D7 of the follow-up and remained hospitalized for treatment with fluid restriction. Interestingly, there was a higher tendency, albeit not significant, for patients having suffered from AVP-D to develop SIAD later on compared to normonatraemic patients at D1. They were then discharged without further complication, and none developed permanent AVP-D during the follow-up. General information regarding the two groups (normonatraemic and AVP-D patients) can be found in Table 1 (see Table 1).

**Table 1:** Comparison of AVP-D and normonatraemic patients. Continuous values are expressed as median and quartiles (Q1; Q3)

	Normonaetremic (n = 54)	AVP-D (n = 13)
Age (y.o.)	60 (48; 68.3)	51 (345.5; 57.5)
Gender (Female)	51.85%	69.2%
Duration of hospitalization after surgery (days)	8 (7; 12)	7 (7; 8)
Preoperative corticoid replacement therapy (yes; no)	11; 43	2; 11
Psychoactive medication (yes; no)	11; 43	1; 12
Pathology (adenoma; other)	49; 5	13; 0
Hormonal secretion from adenoma (yes; no)	14; 40	5; 8
Postoperative basal cortisol (nmol/l)	339 (226; 439)	301 (157; 373)
Hypocortisolemia necessitating maintained corticoid replacement therapy (yes; no)	36; 18	11; 2
Developed SIAD later on (yes; no)	14; 40	6 ; 7
Daily urinary output volume (ml)	888 (639; 1135)	1226 (870; 1796)

### Evolution of oxytocin secretion in the whole group of patients

**Fig. 1:**
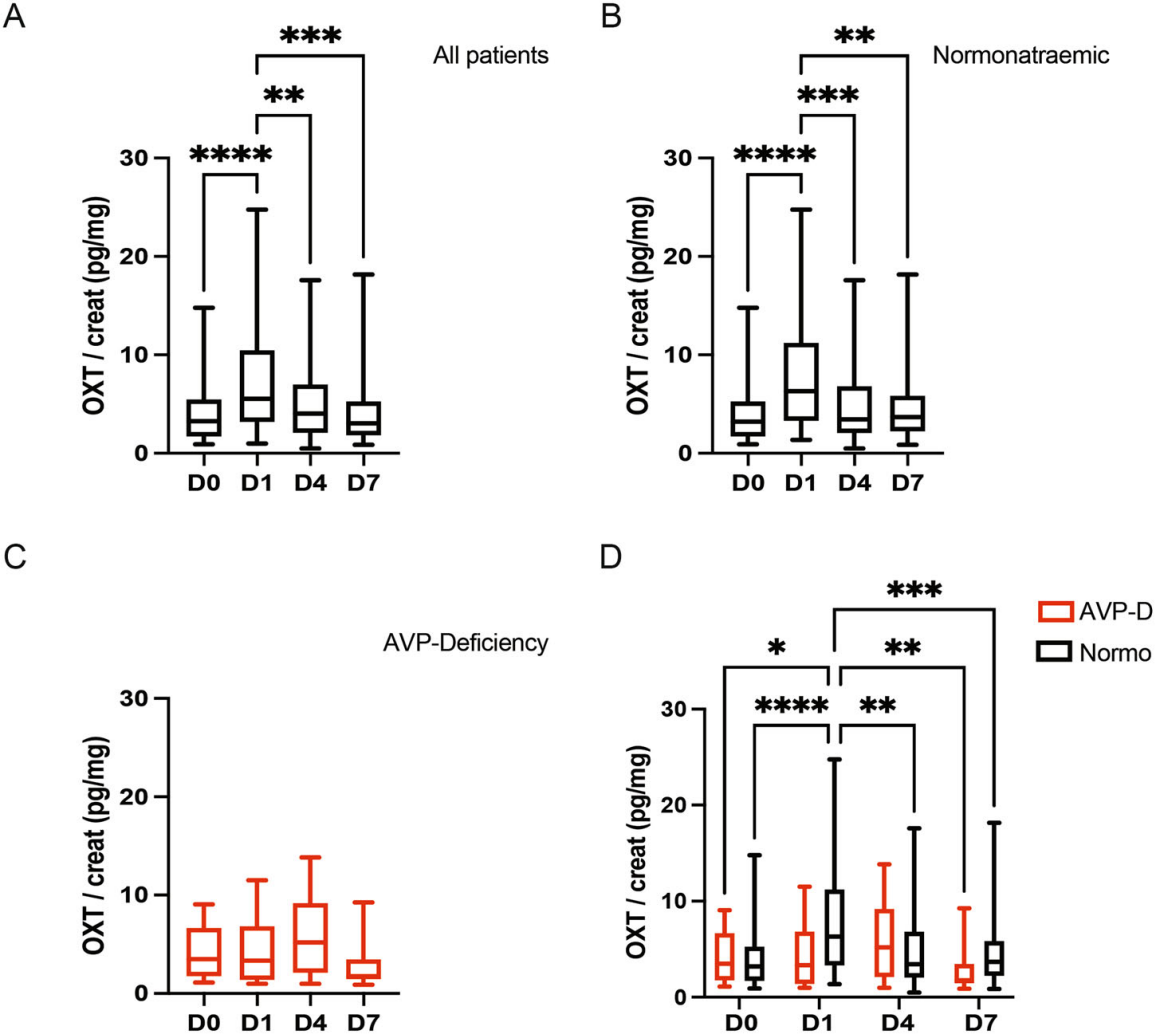
Urinary oxytocin levels show a significant increase between D0 and D1 which is absent in patients developing postoperative AVP-D. **A** Evolution of OXT urinary secretion in all patients between D0, D1, D4, and D7 (n = 67 patients). **B** Evolution of OXT urinary secretion between D0, D1, D4, and D7 in normonatraemic patients (n = 54 patients). **C** Evolution of OXT urinary secretion between D0, D1, D4, and D7 in AVP-D patients (n = 13 patients). **D** Comparison of OXT urinary secretion between both groups (normonatreamic (in black): n = 54 patients, AVP-D (in red): n = 13 patients). Repeated measures one-way ANOVA with Bonferroni post-test (**A**–**C**) and two-way ANOVA with Bonferroni post-test (**D**); Median and quartiles; *p < 0.05, **p < 0.01, ***p < 0.001, ****p < 0.0001

We measured the urinary secretion of OXT in our patients at D0, D1, D4, and D7 after TPS and standardized it with urinary creatinine levels measured concomitantly, i.e. in the morning. When OXT values of all patients were pooled together, OXT secretion showed a significant increase at D1 after TPS when compared to D0. OXT values then returned to preoperative levels at D4 and D7 (see Fig. 1A and Table 2).

**Table 2:** Summary and comparison of urinary OXT values between time-points (median and quartiles (Q1; Q3))

	D0 (pg/mg creat)	D1 (pg/mg creat)	D4 (pg/mg creat)	D7 (pg/mg creat)	p-value (between D0 and D1)	p-value (between D1 and D4)	p-value (between D4 and D7)
All patients together (n = 67)	2.27 (1.73; 5.34)	5.53 (3.21; 10.15)	4.03 (2.1; 6.87)	3.03 (1.85; 5.19)	<0.0001****	0.0063**	>0.999
Normonatraemic patients (n = 54)	3.22 (1.73; 5.14)	6.31 (3.32; 10.97)	3.45 (2.07; 6.71)	3.68 (2.25; 5.59)	<0.0001****	0.0003***	>0.999
AVP-D patients (n = 13)	3.48 (1.88; 6.16)	3.33 (1.59; 5.39)	5.2 (2.13; 8.23)	1.75 (1.5; 3.22)	>0.999	>0.999	0.2526

Repeated measures one-way ANOVA with Bonferroni post-test (A–C) and two-way ANOVA with Bonferroni post-test (D); Median and quartiles

**p < 0.01, ***p < 0.001, ****p < 0.0001

Statistically significant results are presented in bold

### The peak of urinary oxytocin secretion occurs later in patients developing transient arginine vasopressin deficiency

**Fig. 2:**
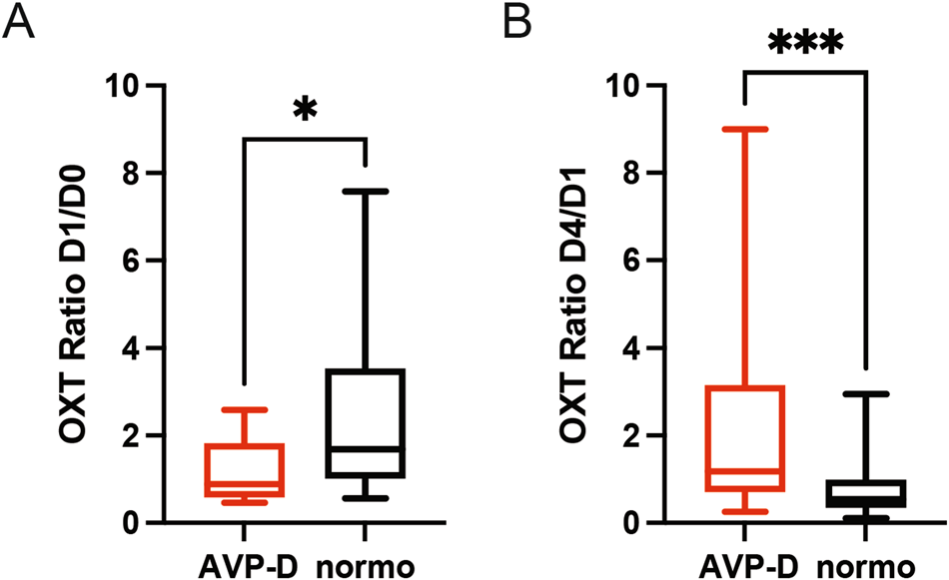
Urinary oxytocin secretion peaks later in AVP-D patients compared to normonatraemic patients. **A** Comparison of the ratio of OXT urinary secretion between D0 and D1 (i.e. the value on D1 relative to the value on D0) in both groups (normonatraemic (in black): n = 54 patients, AVP-D (in red): n = 13 patients). **B** Comparison of the ratio of OXT urinary secretion between D1 and D4 (i.e. the value on D4 relative to the value on D1) in both groups (normonatraemic (in black): n = 54 patients, AVP-D (in red): n = 13 patients). Unpaired Student T-test (**A**, **B**); Median and quartiles; *p < 0.05, ***p < 0.001

We then compared the evolution of the urinary secretion of OXT between patients remaining normonatraemic and patients developing AVP-D after TPS. Interestingly, while the patients remaining normonatraemic at D1 showed a significant peak in OXT secretion at D1, with normalization of OXT secretion at D4, OXT secretion in patients diagnosed with AVP-D did not show a significant increase between D0 and D1, explaining the significant difference in OXT values at D1 between both groups (see Fig. 1B–D and Table 3). Moreover, urinary OXT secretion in the AVP-D group seemed to increase later, namely at D4, although these results failed to reach statistical significance (see Fig. 1C, D and Table 3). These observations were corroborated by comparing the ratios of OXT urinary secretion, between D0 and D1 and between D1 and D4, between the two groups. While the ratio between D0 and D1 was significantly higher in the normonatraemic group compared to the AVP-D group, the ratio between D1 and D4 showed an opposite behavior, with a significantly higher ratio in the AVP-D group compared to the normonatraemic group (see Fig. 2A, B and Table 4).

**Table 3:** Summary and comparison of urinary OXT values between normonatraemic and AVP-D patients (median and quartiles (Q1; Q3))

	Normonatraemic patients (n = 54)	AVP-D patients (n = 13)	p-value (between normonatraemic and AVP-D)
D0 (pg/mg creat)	3.22 (1.73; 5.14)	3.48 (1.88; 6.16)	0.9885
D1 (pg/mg creat)	6.31 (3.32; 10.97)	3.33 (1.59; 5.39)	0.0389*
D4 (pg/mg creat)	3.45 (2.07; 6.71)	5.2 (2.13; 8.23)	0.3667
D7 (pg/mg creat)	3.68 (2.25; 5.59)	1.75 (1.5; 3.22)	0.1043

Unpaired t-test; *p < 0.05

Statistically significant results are presented in bold

**Table 4:** Comparison of OXT dynamics between AVP-D and normonatraemic patients (median and quartiles (Q1; Q3))

	OXT in normonatraemic patients (n = 54)	OXT in AVP-D patients (n = 13)	p-value
OXT ratio D1/D0	1.68 (1.01; 3.51)	0.88 (0.69; 1.36)	0.0162*
OXT ratio D4/D1	0.53 (0.35; 0.92)	1.17 (0.76; 2.82)	0.0006***

Unpaired t-test; *p < 0.05, ***p < 0.001

Statistically significant results are presented in bold

### Absence of normalization of urinary OXT secretion at D4 predicts SIAD

**Fig. 3:**
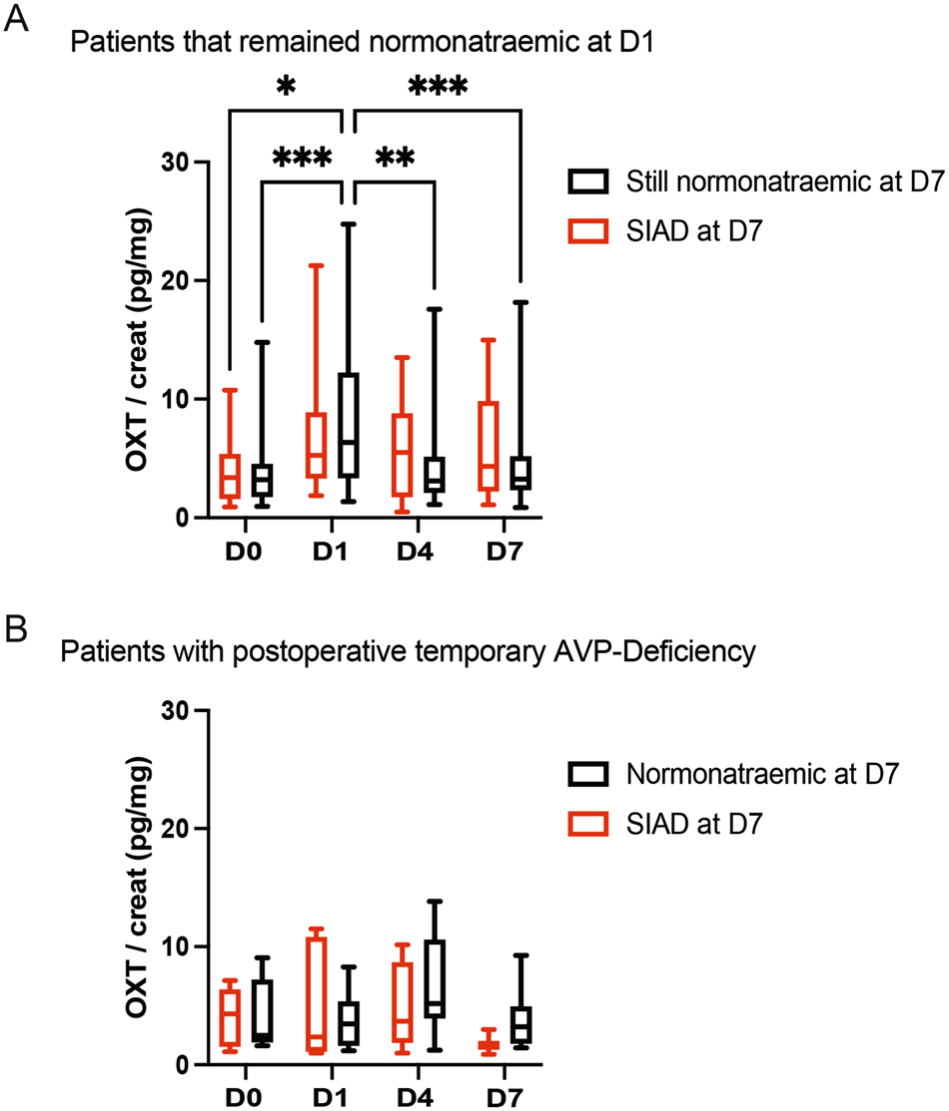
Patients developing SIAD at D7 show a lower peak of OXT urinary secretion at D1. **A** Comparison of OXT urinary secretion in the D1-normonatraemic group (n = 54 patients) between patients remaining normonatraemic (in black, n = 40) and patients developing SIAD (in red, n = 14) at D7. **B** Comparison of OXT urinary secretion in the AVP-D group (n = 13 patients) between patients being normonatraemic (in black, n = 7) and patients developing SIAD (in red, n = 6) at D7. Two-way ANOVA with Bonferroni post-test (**A**, **B**); Median and quartiles; *p < 0.05, **p < 0.01, ***p < 0.001

We then further analyzed the evolution of urinary OXT secretion in the group of patients that did not develop AVP-D after TPS. At D7, there was no statistically significant difference in OXT values between patients remaining normonatraemic and patients developing SIAD (see Table 5). However, patients that remained normonatraemic during the entire hospital stay presented a significant decrease of OXT urinary secretion at D4 compared to D1 after TPS, leading to a normalization of its secretion (compared to preoperative levels). Interestingly, there was no such normalization of OXT secretion at D4 in patients that later developed SIAD or in patients having previously developed AVP-D (see Fig. 3A, B and Table 6).

**Table 5:** Summary and comparison of urinary OXT values (median and quartiles (Q1; Q3)) in the patients that did not present with AVP-D

	Normonatraemic patients during entire hospital stay (n = 40)	Normonatraemic patients at D1 that developed SIAD (n = 14)	p-value
D0 (pg/mg creat)	3.22 (1.75; 4.18)	3.39 (1.67; 5.27)	>0.999
D1 (pg/mg creat)	6.35 (3.33; 12.11)	5.27 (3.68; 7.89)	>0.999
D4 (pg/mg creat)	3.11 (2.09; 5.07)	5.51 (1.81; 8.18)	>0.999
D7 (pg/mg creat)	3.26 (2.35; 5.16)	4.33 (2.23; 9.02)	0.5235

Two-way ANOVA with Bonferroni post-test analysis

**Table 6:** Summary and comparison of urinary OXT values (median and quartiles (Q1; Q3)) in AVP-D patients

	AVP-D patients normonatraemic at D7 (n = 7)	AVP-D patients that developed SIAD (n = 6)	p-value
D0 (pg/mg creat)	2.48 (1.94; 5.36)	4.3 (2.12; 5.88)	>0.999
D1 (pg/mg creat)	3.47 (2.46; 5.13)	2.37 (1.26; 8.71)	>0.999
D4 (pg/mg creat)	5.2 (4.23; 8.71)	3.69 (2.12; 7.48)	>0.999
D7 (pg/mg creat)	3.22 (2.2; 4.33)	1.57 (1.42; 1.71)	0.9521

Two-way ANOVA with Bonferroni post-test analysis

## Discussion

In this study, we measured urinary OXT secretion after TPS in 67 patients with pituitary lesions. We observed that OXT secretion increases later in patients diagnosed with transient AVP-D requiring DDAVP treatment compared with patients showing no AVP-D, suggesting a delay in the release, and then normalization, of OXT after TPS in early, transient AVP-D. We also retrieved that normonatraemic patients in the initial postoperative period, but that later developed SIAD at D7, failed to attenuate and normalize OXT secretion at D4, further supporting the importance for a quick normalization of OXT secretion after its peak release in an uncomplicated postoperative course. Postoperative water and sodium imbalances are common after TPS, occurring in up to three quarters of the cases [35], and encompass many entities such as AVP-D (both as polydipsic or adipsic), postoperative fluid overload, osmotic diuresis and other disorders. Despite the high frequency of their occurrence, diagnosis and, most importantly, differentiation between these clinical conditions remains difficult and based solely on clinical observations and laboratory analyzes. This situation, however, can result in delays, and possible mistakes, in their diagnosis and treatment, which can harm patients. To avoid such complications, recent research has been aimed at facilitating diagnosis of postoperative water-sodium imbalance [36–39], especially AVP-D [9–15, 17, 18, 40, 41]. Postoperative transient AVP-D is known to be caused by a temporarily deficient AVP secretion through the posterior pituitary gland. It manifests with polyuria, decreased urinary specific gravity (USG) and increased sodium natraemia and osmolality [3, 7, 16]. Diagnosis of transient AVP-D has been rendered difficult due to the use of different cut-offs between neurosurgical centers, explaining the wide range of transient AVP-D incidence reported in the literature (2–54%). To homogenize this vast heterogeneity, De Vries et al. proposed standardized criteria to diagnose AVP-D [3]. Nevertheless, diagnosis of AVP-D remains based on several clinical observations and laboratory analyzes set at different time-points, with intervals of several hours between each other. This underlines again the need for early, precise biomarkers of postoperative AVP-D.

In the recent years, copeptin, a byproduct of AVP production that shows a longer half-life and is therefore easier to measure in the plasma, has emerged as a potential biomarker to differentiate between AVP-D and other pathologies of the water-sodium balance [9–12, 14, 15, 40, 41]. In a previous multicentric, prospective study, Winzeler et al. measured copeptin levels before and after TPS and observed an increase in copeptin levels just after TPS. Interestingly, patients exhibiting lower copeptin levels one day after TPS were more likely to develop AVP-D compared to patients remaining normonatraemic [11]. In our study, we also observed an overall significant increase in the secretion of a hormone derived from the posterior pituitary gland, OXT, immediately after TPS. This observation suggests that OXT secretion, which has been described to happen through the same anatomical pathways as AVP [8], might follow the same dynamics as AVP secretion after TPS. Such suggestion is further reinforced by the observations of Atila et al. who identified a deficiency in OXT secretion in patients suffering from central AVP-D [28]. This raises the hypothesis of the existence of a regulatory mechanism (possibly driven by either plasma osmolality or volume) acting on the posterior pituitary and leading to the adaptation of its secretion in response to the operative stress of TPS by increasing hormonal release, possibly to prevent water-sodium imbalance. The inability of patients developing transient AVP-D to increase OXT secretion at D1 found in our study (similar to copeptin secretion in the study of Winzeler et al. [11]) might reflect the failure of such regulatory mechanism, accompanying the development of water-sodium imbalance, namely AVP-D in this case. Some postoperative pituitary stunning may explain the smallest increase of OXT on D1 in the AVP-D group with a delayed release (possibly due to the passive release of both AVP and OXT from degenerating neurohypophyseal nerve terminals in the posterior hypophysis), thereafter peaking on D4, which can be viewed as following the first two phases of the classical triphasic course of AVP-D -SIAD- AVP-D [7, 21]. This is also suggested by the fact that patients that developed transient AVP-D also tended to develop SIAD more often later on (albeit not statistically significant). A question remaining is the potential influence of DDAVP treatment on OXT secretion in DDAVP patients. However, as the abnormal secretion of OXT was more likely to reflect a primary mechanically-induced postoperative dysfunction of the posterior hypothalamus-hypophysis axis (rather than impaired hormonal response to stimulation), the administration of 1 or 2 doses of DDAVP to patients presenting with AVP-D is unlikely to have influenced the whole dynamics of OXT secretion in our patients. We had no permanent AVP-D in our group of patients and therefore cannot draw any conclusion between the course of OXT and persisting AVP-D, a well-known, long-term complication of TPS [20].

Another point of note seems to be the importance of a rapid normalization of OXT secretion after its peak at D1. Indeed, when further analyzing the dynamics of OXT secretion after D1, we observed that patients that developed SIAD later on failed to show normalization of OXT, i.e. a significant decrease, relative to D1, of OXT at D4 and D7. Indeed, in this patients’ subgroup, OXT secretion seemed (albeit results failed to reach statistical significance) to still show a positive slope between D1 and D4 compared to a negative slope for the patients that remained normonatraemic at D7 (see Fig. 3). This observation appears to be consistent with our previous publication in which we already reported an abnormal elevation of OXT secretion on D4 compared to D1 in patients developing SIAD [30] and further reinforces the potential for this hormone as an early biomarker of postoperative dysnatraemia. Finally, as a relative increase (or lack of normalization) of OXT release on D4 might portend SIAD, a lack of increase on D1 might presage of early AVP-D and help in its diagnosis. The question, whether dysregulation of OXT secretion might significantly contribute to the occurrence of the postoperative dysnatraemia or is simply an epiphenomenon reflecting anatomical disruption between neighboring systems still remains open and warrants further study. Indeed, while reports have suggested that OXT cross-reacts with AVPR2 and therefore contributes to antidiuresis [42], the later will likely correlate primarily with AVP secretion as this pathway remains the principal regulator of water reabsorption, particularly due to its sensitivity to osmotic stimulation. While OXT measurement, like AVP, remains complicated [43, 44] and complete correlation between central (measured in the cerebrospinal fluid (CSF)) and peripheral (measured in the blood and urine) OXT levels has not been shown [44, 45], these results still give weight to a role for OXT in postoperative dysnatraemia and underlined its potential in the prediction of the aforementioned dysnatraemia. Moreover, there is still a need to develop faster, and more robust, methods for the testing of OXT levels, both in urine and in the blood, to take full advantage of this hormone’s capability as an early biomarker of postoperative dysnatraemia [43, 45]. The variability in the range of urinary OXT secretion measured in our study points out this lack. Nevertheless, the fact that the basal urinary OXT values measured in our study were in line with the ones reported in the literature [33, 34] and the use of OXT ratios and repeated measures in our comparisons mitigates the effect of such variability on our results. In light of these considerations, the potential of OXT measurement as a biomarker not only of SIAD, but possibly also of AVP-D, appears more clearly.

Lastly, previous reports suggested a role for OXT in the regulation of the renine-angiotensine-aldosterone pathway, a well-known regulatory pathway of water-sodium balance [46, 47], and in the regulation of glucose and lipids metabolism [48–51]. Furthermore, OXT has also been implicated in the regulation of interpersonal relationships and humans’ behavior [52–57]. The identification of such an abnormal OXT secretion after TPS might therefore be of clinical relevance as it would warrant the postoperative evaluation of those different physiological pathways. This also applies for the effects of OXT on behavior as postoperative stress and anxiety could be correlated with OXT levels, possibly as an adaptive mechanism. This could perhaps lead to explore the potential use of intranasal OXT as a treatment for post-surgical stress [58–60].

Our study is not devoid of limitations. We only measured OXT secretion in the urine and not in the plasma or CSF of our patients. As full correlation between central and peripheral OXT concentrations has been previously questioned [45, 58], further research is required to evaluate the correlation of OXT secretion between urine, plasma and CSF. Of note, Francis et al. previously reported the occurrence of a correlation between plasmatic and urinary OXT levels [33]. The use of different analytical methods would also be of interest. Indeed, while OXT was tested using radioimmunoassay in this study, ELISA testing might be more appropriate for clinical use due to its superior speed and lower cost. Interestingly, Gnanadesikan et al. [61] recently reported a good specificity for commercially available immunoassays measuring OXT levels in the plasma of mice. They also observed that unextracted samples gave larger concentration values. Moreover, we did not measure copeptin levels in the plasma of our patients, as it was not an objective of this study. According to the results of Winzeler et al. [11], a dynamic of the secretion of copeptin similar to the one of OXT might have been expected in our patients. Whether the performance of any of those two biomarkers is superior to the other one remains however to be determined. Further research, in which both OXT and copeptin secretion are concomitantly measured, is therefore warranted to 1) compare the secretion of both hormones with each other, 2) confirm the observed dynamics of those hormones after TPS, 3) establish whether either copeptin or OXT might be superior in the diagnosis of AVP-D after TPS and 4) study the potential of a combined measure of those hormones in the diagnosis of postoperative dysnatraemia. Finally, as none of our patients developed permanent AVP-D, we were not able to compare the evolution of OXT in this condition and further research is needed to 1) analyze the evolution of OXT secretion in patients developing permanent AVP-D and 2) evaluate whether OXT secretion might help to identify patients later developing permanent AVP-D. Finally, a potential role for OXT secretion in the observed dysnatraemia was also not directly tested and warrants further, prospective studies.

## Conclusion

In this study, we show for the first time that urinary OXT secretion increases later in patients developing transient AVP-D. We also observe that a rapid normalization of OXT after its peak is normal, and that patients later developing SIAD fail to normalize OXT secretion. This observation further reinforces the potential for OXT as a biomarker for postoperative dysnatraemia.

## Data Availability

Data are available from corresponding author upon reasonable request.
